# Ectopic Sphenoid Sinus Prolactinoma Associated With Empty Sella and Recurrent Meningitis Treated Successfully With Cabergoline

**DOI:** 10.7759/cureus.63058

**Published:** 2024-06-24

**Authors:** Winston Y Chang, Kevin Spitler, Gilbert Cheung, Brandon Chock

**Affiliations:** 1 Internal Medicine, Kaiser Permanente Fontana Medical Center, Fontana, USA; 2 Radiology, Kaiser Permanente Fontana Medical Center, Fontana, USA; 3 Endocrinology, Diabetes, and Metabolism, Kaiser Permanente Fontana Medical Center, Fontana, USA

**Keywords:** cabergoline, empty sella, meningitis, ectopic pituitary adenoma, pituitary adenoma, ectopic prolactinoma

## Abstract

Ectopic pituitary adenomas (EPA) are tumors that reside outside the sella turcica and are not connected to the pituitary gland. We present a case of a 62-year-old female who was hospitalized for recurrent meningitis. Workup, including magnetic resonance imaging (MRI) of the brain, computed tomography (CT) of the sinuses, and follow-up MRI of the sella turcica, revealed an 8 millimeter (mm) mass in the right posterior sphenoid sinus with an empty sella turcica and no evidence of a sellar mass. The mass was biopsied, with pathology results showing findings of a pituitary adenoma staining positive for prolactinoma. Subsequent hormonal workup revealed an elevated prolactin level of 128.4 nanograms per milliliter (ng/mL) without evidence of additional hormonal co-secretion or hypopituitarism. The patient was started on cabergoline with eventual normalization of the prolactin level and regression of the adenoma on follow-up imaging. EPAs are rare and thus treatment guidelines have not been established. Our case report not only highlights a case of successful treatment of ectopic prolactinoma with dopamine agonist therapy but also calls attention to the uncommon co-existence of EPAs and empty sella turcica and meningitis as an extremely uncommon presentation of EPAs.

## Introduction

Pituitary adenomas account for 10-15% of intracranial tumors [1]. Most pituitary adenomas present within the sella turcica; however, there are rare cases of ectopic pituitary adenoma (EPA) that occur when an adenoma resides exclusively outside of the sella turcica, whether it be in intra-cranial or extra-cranial locations [2]. Approximately 40% of EPAs are located in the sphenoid sinus [3]. These EPAs may also uncommonly present with an empty sella (thought to be related to a developmental disorder of anterior pituitary tissues [4]) or with meningitis (secondary to retrograde transmission of bacteria through a bony defect in the sphenoid sinus). Here, we present a case of ectopic sphenoid sinus prolactinoma associated with both empty sella and recurrent meningitis that was treated successfully with cabergoline.

## Case presentation

**Figure 1:**
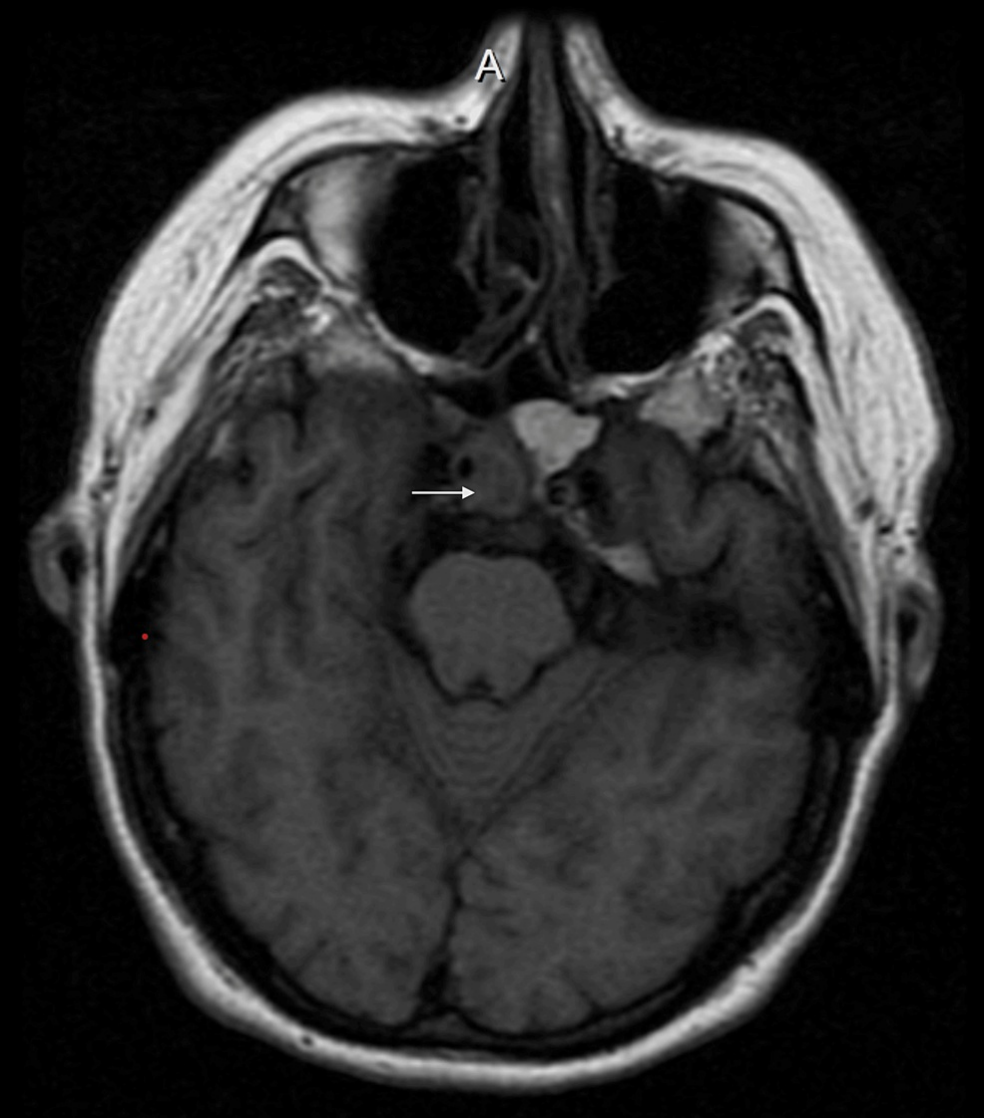
MRI brain showing opacification in bilateral sphenoid sinuses

A 62-year-old female with a history of Streptococcus pneumoniae meningitis two years prior, hypertension, and obesity presented with headache, fevers, chills, and confusion. Lumbar puncture again revealed Streptococcus pneumoniae meningitis and the patient was started on antibiotics with clinical improvement. An initial MRI of the brain revealed bilateral sphenoid sinusitis without evidence of brain abscess (Figure 1).

**Figure 2:**
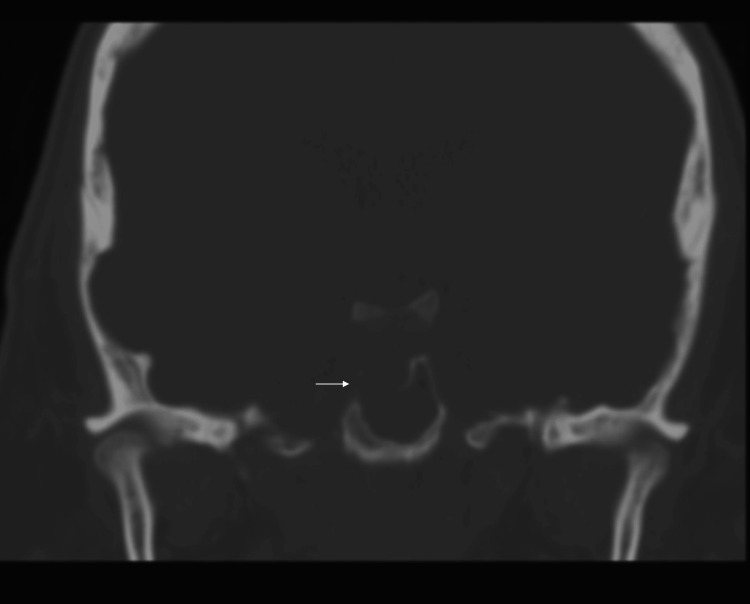
CT sinus showing apparent destruction of the right sellar floor

Subsequent CT of the sinus showed extensive sphenoid sinus disease, with apparent destruction of the right sellar floor (Figure 2).

**Figure 3:**
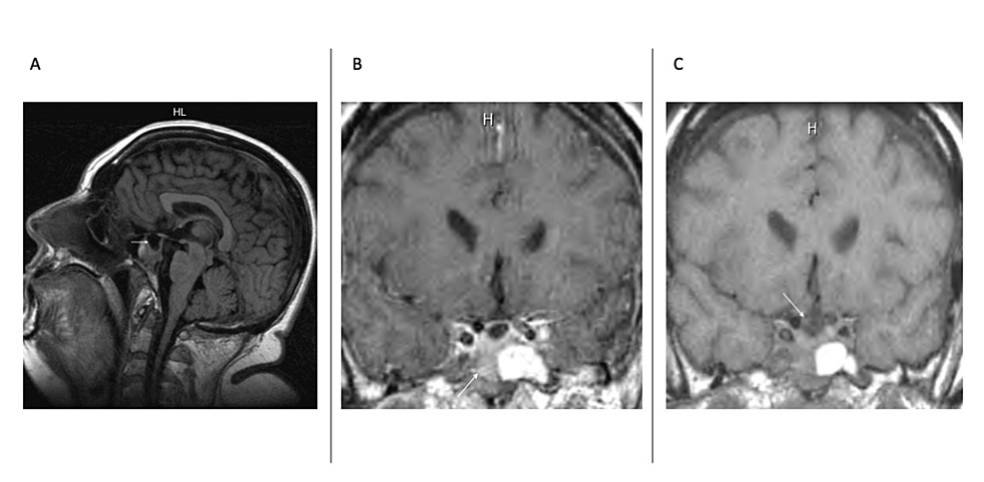
MRI of the sella turcica showing an empty sella (A), a mass in the right posterior sphenoid sinus (B), and no evidence of a sellar mass (C)

Follow-up MRI of the sella turcica revealed an empty sella (Figure 3A) and an 8-millimeter mass in the right posterior sphenoid sinus (Figure 3B) without evidence of a sellar mass (Figure 3C).

Due to her recurrent episodes of meningitis, the patient underwent endoscopic sphenoidotomy, confirming an abnormally expanded area below the sella seen on her previous imaging. A biopsy of the sphenoid sinus mass revealed a pituitary adenoma that stained positive for prolactin.

**Figure 4:**
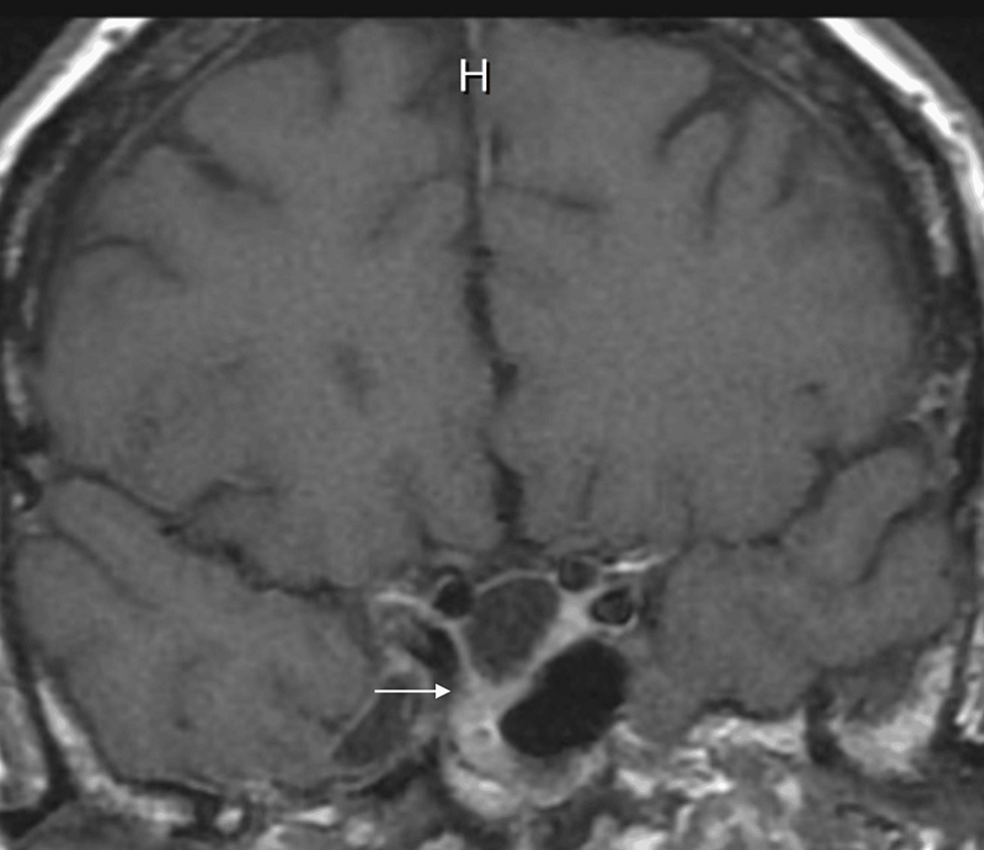
MRI of the sella turcica showing regression of adenoma in the right sphenoid sinus

Hormonal studies revealed elevated serum prolactin (PRL) at 128.4 ng/mL. There was no evidence of additional hormonal co-secretion or hypopituitarism. At the time of the initial endocrine evaluation, the patient denied any headaches, vision changes, or galactorrhea. She had been having regular periods prior to a hysterectomy at age 58. There was no family history of endocrine disorders. The patient was initiated on cabergoline 0.5 mg once weekly with excellent response and normalization of PRL to 4.7 ng/mL after two months. Follow-up MRI 18 months later showed regression of the adenoma to 4 mm (Figure 4).

Over the next six years, as prolactin levels remained within normal limits and the patient remained asymptomatic with stable findings on MRI, cabergoline was gradually decreased to 0.25 mg every other week. The patient was given the option of further tapering off the cabergoline but elected to remain on the current dosage. Unfortunately, the patient was lost to follow-up, as she was found to have metastatic adenocarcinoma of unknown origin to the liver and pancreas and elected to pursue hospice.

## Discussion

EPAs are extremely rare tumors, with only approximately 100 cases reported in the English literature [5]. By definition, they are extrasellar pituitary adenomas that demonstrate no connection to the pituitary gland. They are challenging to diagnose, as they must first be distinguished from invasive pituitary adenomas, which originate in the sella turcica but expand into the sphenoid sinus with destruction of the sellar floor. The distinction between these two types of pituitary adenomas can be made based on the state of the dura of the sellar floor, whether it is intact or damaged. These findings can be accurately seen on MRI, but ultimately, the gold standard for evaluating this distinction is surgical proof of an intact dura. In addition, empty sella may be associated with EPAs. Only a total of 15 cases of co-existence of an ectopic pituitary adenoma and empty sella were reported in a recent review by Liang et al. [6]. In our patient who was successfully treated with medical therapy, surgery was deferred and thus we were unable to obtain surgical proof of an intact dura. However, the findings of empty sella and the absence of a sellar mass on the initial MRI would suggest that an invasive pituitary adenoma would be unlikely, and the adenoma most likely originated from the sphenoid sinus.

Treatment guidelines for ectopic prolactinomas have not been clearly established due to their scarcity. For the treatment of symptomatic prolactinoma, Endocrine Society guidelines generally recommend initial treatment with dopamine agonists to lower prolactin levels and decrease tumor size [7]. Transsphenoidal surgery is offered to symptomatic patients who cannot tolerate or do not respond to medical therapy. To our knowledge, there exists only one other case report of an ectopic prolactinoma in the sphenoid sinus with empty sella syndrome [8]; however, the patient did not respond to dopamine agonist therapy and ultimately underwent surgical treatment. Our patient was started on cabergoline with eventual normalization of prolactin levels as well as tumor size regression from 8 mm to 4 mm on follow-up MRI 18 months after presentation.

Our case also highlights an extremely uncommon clinical presentation of EPAs. Meningitis has been noted to be an unusual presentation of an invasive pituitary adenoma, which occurs usually due to infection of the cerebrospinal fluid (CSF) leaking through the erosion of the wall of the sphenoid sinus and thus allowing entry of nasopharyngeal organisms [9]. EPAs from the sphenoid sinus eroding through the bony wall of the sphenoid sinus and presenting as meningitis had not been reported in the literature until a recent case report in 2018 by Akinduro et al. [10]. In the case report, the patient was later found to have ectopic prolactinoma. Our patient presented similarly with recurrent meningitis due to erosion of the sellar floor in the setting of an ectopic sphenoid sinus prolactinoma and was also noted to have findings of empty sella, which is even more unique. In our case, successful treatment of the patient's EPA resulted in clinical improvement by preventing the recurrence of meningitis.

## Conclusions

In summary, this case emphasizes the challenges in diagnosing EPAs and differentiating them from invasive pituitary adenomas. Treatment guidelines for ectopic prolactinomas are not well established but usually, medical treatment should be attempted first in the absence of clear surgical indications. Furthermore, our case sheds light upon an unusual presentation for EPAs; though rare, they should always be considered in patients presenting with an empty sella as well as in the differential diagnosis in patients presenting with meningitis with clinical or radiographic evidence of sinusitis.
